# Dr. Harry M. Hallock

**Published:** 1913-08

**Authors:** 


Dr. Harry M. Hallock, P. & S. (Columbia), 1889, Major in the Medical Corps, U. S. A., was found dead in the woods near Hot Springs Ark., May 19, having shot himself in the head. He was 45 years old. He was retired in 1908 on account of disabilities received in the service and was, later, for some time Medical Director of the Government Reservation at Hot Springs. Major Hallock was, several years ago, stationed at Fort Porter, Buffalo, and was well known to many of the local profession.

